# STAT3 imparts *BRCA*ness by impairing homologous recombination repair in Epstein-Barr virus-transformed B lymphocytes

**DOI:** 10.1371/journal.ppat.1008849

**Published:** 2020-10-01

**Authors:** Michael T. McIntosh, Siva Koganti, J. Lucas Boatwright, Xiaofan Li, Salvatore V. Spadaro, Alexis C. Brantly, Jasmine B. Ayers, Ramon D. Perez, Eric M. Burton, Sandeepta Burgula, Thomas MacCarthy, Sumita Bhaduri-McIntosh

**Affiliations:** 1 Child Health Research Institute, Department of Pediatrics, University of Florida, Gainesville, FL, United States of America; 2 Department of Molecular Genetics and Microbiology, University of Florida, Gainesville, FL, United States of America; 3 Division of Infectious Diseases, Department of Pediatrics, Stony Brook University, Stony Brook, NY, United States of America; 4 Bioinformatics Core Facility, University of Florida, Gainesville, FL, United States of America; 5 Division of Infectious Diseases, Department of Pediatrics, University of Florida, Gainesville, FL, United States of America; 6 Department of Microbiology and Immunology, Stony Brook University, Stony Brook, NY, United States of America; 7 Laufer Center for Physical and Quantitative Biology, Stony Brook University, Stony Brook, NY, United States of America; Kansas State University, UNITED STATES

## Abstract

Epstein-Barr virus (EBV) causes lymphomas and epithelial cell cancers. Though generally silent in B lymphocytes, this widely prevalent virus can cause endemic Burkitt lymphoma and post-transplant lymphoproliferative disorders/lymphomas in immunocompromised hosts. By learning how EBV breaches barriers to cell proliferation, we hope to undermine those strategies to treat EBV lymphomas and potentially other cancers. We had previously found that EBV, through activation of cellular STAT3 prevents phosphorylation of Chk1, and thereby, suppresses activation of the intra-S phase cell-cycle checkpoint, a potent barrier to oncogene-driven proliferation. This observation prompted us to examine the consequences on DNA repair since homologous recombination repair, the most error-free form, requires phosphoChk1. We now report that the defect in Chk1 phosphorylation also curtails RAD51 nucleation, and thereby, homologous recombination repair of DNA double strand breaks. The resulting reliance on error-prone microhomology-mediated end-joining (MMEJ) repair makes EBV-transformed cells susceptible to PARP inhibition and simultaneous accrual of genome-wide deletions and insertions resulting from synthesis-dependent MMEJ. Analysis of transcriptomic and drug susceptibility data from hundreds of cancer lines reveals a STAT3-dependent gene-set predictive of susceptibility of cancers to synthetic lethal PARP inhibition. These findings i) demonstrate how the tumor virus EBV re-shapes cellular DNA repair, ii) provide the first genome-wide evidence for insertions resulting from MMEJ in human cells, and iii) expand the range of cancers (EBV-related and -unrelated) that are likely to respond to synthetic lethal inhibitors given the high prevalence of cancers with constitutively active STAT3.

## Author summary

In a transformation model based on the cancer-causing Epstein-Barr virus (EBV), we previously demonstrated that the cellular oncoprotein STAT3, activated during transformation, impairs ATR's ability to phosphorylate Chk1 in response to replication stress, thereby relaxing the intra-S phase checkpoint. While this relaxation allows EBV-transformed cells to rapidly traverse the S phase checkpoint, we now show that activation of STAT3 has another important consequence. Specifically, impaired Chk1 phosphorylation results in impaired homologous recombination repair, a form of high-fidelity repair in replicating cells. As a result, EBV-transformed proliferating cells become dependent on error-prone microhomology mediated end-joining (MMEJ) repair, accumulate not only deletions but also genome-wide insertions from such repair, and succumb to inhibition of PARP, an enzyme critical for MMEJ repair. These findings also link STAT3 activation, commonly observed in human cancers, to PARP inhibitor susceptibility, and simultaneously provide a predictive STAT3 gene expression signature.

## Introduction

Epstein-Barr virus (EBV) is causally linked to endemic Burkitt lymphoma in equatorial Africa and B-cell lymphoproliferative diseases (LPD)/lymphomas in immunocompromised individuals such as those with HIV/AIDS, transplant recipients, or individuals on immunomodulatory agents [1–3]. LPD in the setting of therapeutic immunosuppression to prevent graft rejection in solid organ or hematopoietic transplant recipients can be a devastating complication. In the absence of T cell surveillance, EBV-infected B cells can proliferate rapidly, leading to LPD. Therapeutic approaches include reduction of immunosuppression (RIS), ablation of CD20^+^ B cells using Rituximab, adoptive T cell therapies, combination chemotherapy, surgery, and radiation therapy [4–8]. While these improve survival and quality of life of LPD patients, RIS can lead to damage or loss of transplanted organs, Rituximab causes global and often long-term B cell immunodeficiency, T cell therapies are not widely available, and chemotherapy, surgery, and radiation therapies are effective in selected cases, thus highlighting the need for additional strategies for prevention and treatment.

A WHO Group 1 carcinogen, EBV encodes potent oncoproteins that aggressively drive B cell proliferation resulting in immortalized lymphoblastoid cells lines (LCL) in culture. As LCL are an excellent model to study immunocompromise-associated LPD, we are using them to identify strategies that EBV uses to dampen cell-intrinsic barriers to ensure that transformed cells are able to proliferate. Our goal is to then target those strategies to cripple proliferation of transformed/cancer cells. We have shown that EBV oncoproteins drive rapid cellular DNA replication causing DNA forks to stall, and sometimes collapse, resulting in activation of cellular ATR [9, 10]. Our earlier studies have also shown that EBV uses the cellular proto-oncogene STAT3 (phosphorylated at Y705) to block ATR’s ability to phosphorylate Chk1 –ensuring that the intra-S phase checkpoint is sufficiently relaxed to allow transformed cells to progress through the cell cycle[9–11]. With phospho-Chk1 also essential for key functions such as homologous recombination repair (HR) [12, 13], particularly in cancer cells, we investigated the effects of blunted Chk1 phosphorylation on DNA repair in EBV-transformed cells.

We now find that in EBV-infected replicating cells, STAT3-mediated disruption of Chk1 phosphorylation impairs RAD51 nucleation (a key step in HR), HR, and recovery following experimentally imposed DNA double-strand breaks. These HR-impaired proliferating cells not only retain but now rely on error-prone microhomology-mediated end-joining (MMEJ)-mediated repair, resulting in susceptibility to PARP inhibition; PARP1 is known to be required for MMEJ-mediated repair[14]. Importantly, we also show signatures of MMEJ-induced deletions as well as small and large insertions in the genomes of EBV-transformed human B lymphocytes. Further, by analyzing gene expression profiles of cancer lines derived from a range of tissues, we report a STAT3-dependent gene set that is predictive of susceptibility to PARP inhibition of blood and other types of cancer. These findings not only reveal that EBV-transformed cells are susceptible to PARP inhibitors and the mechanism for that susceptibility, but that this susceptibility also likely extends to EBV-unrelated blood and other cancers as STAT3 is constitutively active in about two-thirds of human cancers.

## Results

### STAT3 impairs RAD51 foci formation

**Fig 1:**
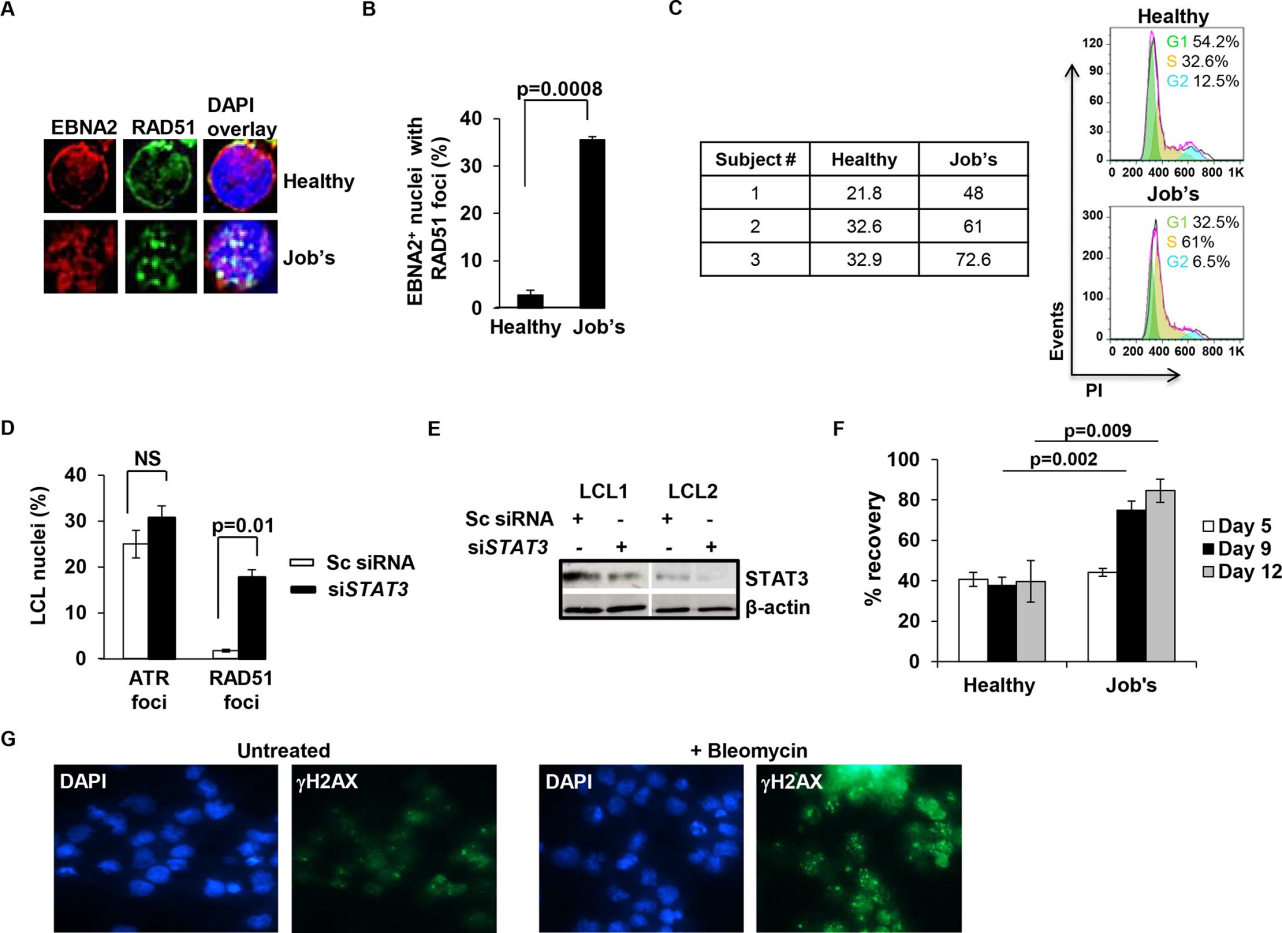
EBV-infected/transformed proliferating B cells with functional STAT3 demonstrate scarce RAD51 foci-containing nuclei. (A and B) Primary B lymphocytes from healthy subjects and patients with Job’s syndrome were infected with EBV and placed in culture for 4 days. Representative immunofluorescence images of nuclei stained with DAPI and for EBNA2 and costained for RAD51 are shown in A. Aggregate data from 100 EBNA2^+^ nuclei each from healthy and Job’s cells are shown in B. Table in C shows percent infected cells in S phase on day 4; cell cycle profiles of representative healthy and Job’s samples are shown on the right. (D and E) Two healthy subject-derived EBV-transformed cell lines (LCL) were transfected with siRNA to STAT3 or scrambled (Sc) siRNA and harvested 36h later. Aggregate data from immunofluorescence images of >100 nuclei stained with DAPI and costained for ATR or RAD51 are shown in D. Cells were subjected to immunoblotting for STAT3 and β-actin in E. (F and G) Bleomycin-treated LCL derived from 3 healthy subjects and 3 Job’s syndrome patients were enumerated for live cells using Trypan blue staining on indicated days and percent recovery calculated by comparing to matched Bleomycin-untreated LCL (F). Immunofluorescence images of representative Bleomycin-exposed LCL nuclei that were costained for DAPI and γH2AX are shown in G; error bars indicate SEM in B, D, and F.

During DNA replication of proliferating cells, cell cycle checkpoints and DNA repair need to be tightly coordinated. Such coordination ensures that cells are not excessively delayed within any phase of the cell cycle yet enough time is allowed for repair of DNA lesions. We had previously shown that EBV-infected/transformed cells experience replication stress as measured by RPA and ATR nuclear foci. However, EBV activates STAT3 to block ATR’s ability to phosphorylate Chk1, thereby suppressing the intra-S phase checkpoint response [10, 11]. Since phosphoChk1 also facilitates BRCA2-mediated RAD51 recruitment to HR repair foci [12, 13], we asked whether RAD51 foci formation was compromised in EBV-infected/transformed cells. Using EBV-infected primary B cells from healthy subjects and patients with Job’s syndrome (in whom the majority of STAT3 is nonfunctional despite normal levels of STAT3 protein) [15, 16], we found that very few (2–3%) infected nuclei marked by EBV EBNA2 staining had RAD51 foci when STAT3 was functional. In contrast, >35% EBNA2^+^ nuclei demonstrated RAD51 foci when STAT3 was impaired (~11 to 17-fold difference between STAT3-intact and STAT3-impaired cells; Fig 1A and 1B).

Notably, there was only a 2-fold difference between percent cells in the S phase in STAT3-intact versus STAT3-impaired cells (Fig 1C), consistent with our previous observation that EBV-infected STAT3-impaired cells arrest in the S phase [9]. In a complementary approach, siRNA-mediated knockdown of STAT3 in EBV-transformed cells (LCL) demonstrated significant recovery of cells with RAD51 foci. However, lack of increase in ATR^+^ cells indicated that STAT3 does not influence replication stress or its detection (Fig 1D and 1E). Furthermore, LCL with functional STAT3 recovered poorly from experimentally imposed DNA double strand breaks (DSBs) compared to LCL with impaired STAT3 (Fig 1F), suggesting that HR aided the recovery of Job’s cells with Bleomycin-induced DSBs. As expected, exposure to Bleomycin resulted in increased γH2AX nuclear foci, indicative of DSBs (Fig 1G). Thus, STAT3 curtails RAD51 nucleation and the cellular response to DSBs in EBV-transformed cells.

### STAT3 limits homologous recombination-mediated DSB repair through Chk1

**Fig 2:**
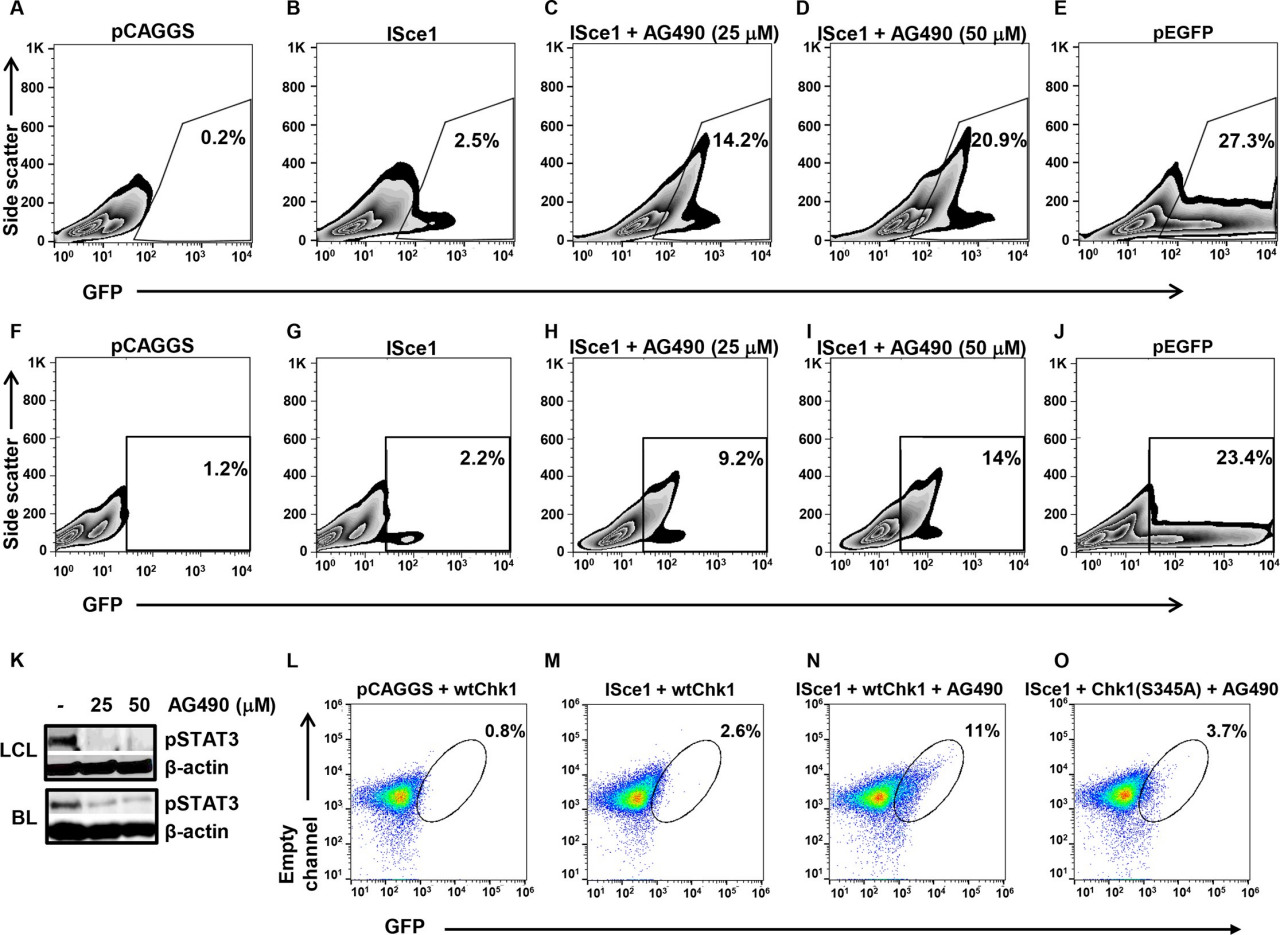
STAT3 restricts HR repair through Chk1 in EBV-transformed cells. (A-K) LCL derived from a healthy subject (A-E, K) and EBV-positive HH514-16 Burkitt lymphoma (BL) cells (F-J, K) were transfected with DR-GFP plasmid (A-D, F-I) and empty vector pCAGGS (A, F) or ISce1 plasmid (B-D, G-I), treated with 25μM (C, H) or 50μM (D, I) AG490 after 18h, and harvested after another 30h for analysis of GFP-positive cells by flow cytometry (A-D, F-I) and immunoblotting for phospho(p)STAT3 and β-actin (K). LCL (E) and BL cells (J) were transfected in parallel with pEGFP to monitor transfection efficiency. (L-O) BL cells with stably-integrated DR-GFP were transfected with Chk1 plasmid (wild-type [L-N] or S345A mutant [O]) and pCAGGS (L) or ISce1 plasmid (M-O), treated with 50μM AG490 after 18h, and harvested after another 30h for analysis of GFP-positive cells by flow cytometry. Numbers in plots indicate percent GFP-positive cells; both side scatter (A-J) and empty channel (L-O) lack fluorescence staining. Experiments were performed 3 times.

To determine if reduction in RAD51 foci-bearing cells indeed reflected poor HR-mediated repair or simply a dearth of DSBs, we tested the ability of EBV-transformed cells and BL cells to repair a defined DSB using a plasmid-based DR-GFP reporter assay [17]. In this assay, HR is indicated by repair of the plasmid and restoration of GFP fluorescence. Both LCL and BL cells showed very few (1–2.3%) repair competent cells despite transfection efficiencies >20% (Fig 2A, 2B, 2E, 2F, 2G and 2J). Furthermore, in the presence of increasing concentrations of AG490, a Janus kinase inhibitor that inhibits STAT3 phosphorylation [9, 10, 18], the percentages of GFP^+^ cells simultaneously increased (Fig 2C, 2D, 2H, 2I and 2K).

To address if STAT3 restricted HR-mediated repair via Chk1, we examined AG490-exposed cells for GFP expression in the presence of wild-type versus a phospho-dead (S345A) mutant of Chk1. While STAT3-impaired cells demonstrated HR-mediated DSB repair, repair was limited in the presence of the Chk1 mutant (Fig 2L–2O), indicating that a STAT3-Chk1 axis is responsible for disrupting HR-mediated repair in EBV-transformed cells.

### EBV-transformed cells and EBV-positive Burkitt lymphoma cells exhibit *BRCA*ness

DSBs that result from collapsed replication forks are highly genotoxic if not repaired. Typically, high fidelity repair of such DSBs is mediated by HR during S and G2 phases of the cell cycle. Because cancer is characterized by repeated and often unscheduled rounds of DNA replication, resulting in increased DNA lesions, transformed/cancer cells in particular require efficient DNA repair. Indeed, loss of DNA repair of one type makes cancer cells dependent on other repair mechanisms–and–such cancers are likely to succumb to approaches that interfere with the remaining mechanism(s) of DNA repair. This phenomenon, known as synthetic lethality, is exhibited by cancers with biallelic mutations in HR genes such as *BRCA1* or *BRCA2* [19, 20]. Synthetic lethal agents include PARP inhibitors that are a group of pharmacological inhibitors of the enzyme poly-ADP ribose polymerase. Since HR-deficient cancers depend on other modes of DNA repair including those requiring PARP, inhibition of PARP is detrimental to their survival. This susceptibility of HR-deficient cancers to synthetic lethal approaches is commonly referred to as *BRCA*ness [21, 22].

**Fig 3:**
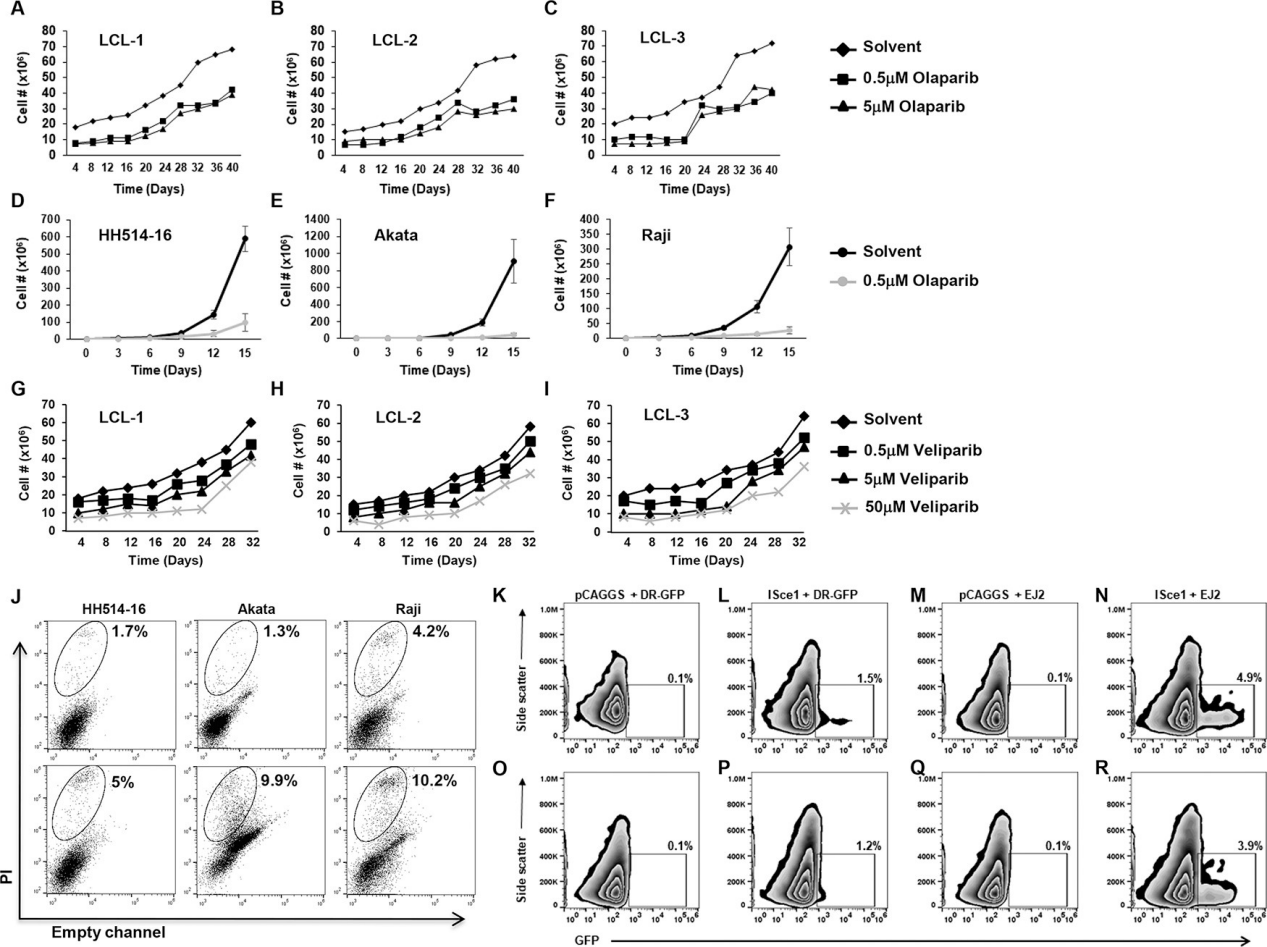
EBV-transformed cells are susceptible to PARP inhibition and demonstrate MMEJ-mediated DSB repair. (A-I) LCL derived from three healthy subjects (A-C and G-I) and three EBV^+^ BL cell lines (HH514-16, Akata, and Raji; D-F) were grown in the presence of Olaparib (A-F) or Veliparib (G-I) and enumerated for live cells on indicated days; PARP inhibitors were added at time 0 and every 3–4 days thereafter at indicated concentrations. (J) EBV^+^ BL cell lines (HH514-16, Akata, and Raji) were left untreated (upper panels) or treated with 0.5μM Olaparib every 3–4 days (lower panels), harvested on day 8, and assayed for dead cells using propidium iodide (PI) staining and flow cytometry. (K-R) LCL (K-N) and HH514-16 BL cells (O-R) were transfected with DR-GFP plasmid (K, L, O, P) and empty vector pCAGGS (K, O) or ISce1 plasmid (L, P) versus EJ2 plasmid (M, N, Q, R) and pCAGGS (M, Q) or ISce1 plasmid (N, R) and harvested after 48h for analysis of GFP-positive cells by flow cytometry. Numbers in plots indicate percent GFP-positive cells. Experiments were performed 3 times.

Given the defect in HR-mediated repair in EBV-transformed cells, we examined the effect of Olaparib and Veliparib, two PARP inhibitors used in the clinic, on several LCL derived from healthy subjects and EBV^+^ BL-derived lines. Though typically used in combination with other anti-cancer agents, Olaparib when used alone in concentrations that have been previously described [23], demonstrated a 50% reduction in growth of LCL (Fig 3A–3C). The effect was more pronounced in BL lines, which exhibited exquisite sensitivity to PARP inhibition (Fig 3D–3F). Similarly, Veliparib used alone in increasing concentrations [24] demonstrated progressive decreases in LCL growth (Fig 3G–3I). Also, compared to solvent, exposure of BL cells to Olaparib led to increased cell death (Fig 3J). This 2.4 to 7.6-fold increase in cell death is consistent with observations that loss of cancer cells following exposure to PARP inhibitors is a gradual and cumulative process and takes several days to weeks. Of note, PARP inhibitors Olaparib and Veliparib do not impair phosphorylation of STAT3 [23]. Thus, EBV-transformed cells and BL lines known to have constitutively active STAT3 [25], demonstrate impaired HR and succumb to synthetic lethal approaches such as PARP inhibition.

### EBV-transformed cells are proficient in MMEJ-mediated DSB repair and harbor large deletions and insertions bearing MMEJ signatures

Impaired HR-mediated repair in the face of oncogene-induced replication stress and reliance on PARP suggested that EBV-transformed cells utilized the error-prone mechanism of MMEJ to repair DSBs. During MMEJ-mediated repair, PARP facilitates recruitment of DNA polymerase theta to DSBs [26]. We therefore tested LCL and BL cells for their ability to perform MMEJ-mediated DSB repair using the EJ2-GFP reporter assay [27] and found that both types of cells utilized MMEJ to repair DSBs (Fig 3K–3R).

MMEJ can result in error-free repair, deletions with microhomologies, and possibly, insertions with or without microhomologies. Given that EBV^+^ cells were MMEJ-competent, we sought evidence for naturally occurring MMEJ-repair in the genomes of newly generated 2-week old LCL. To examine whole genomes, we generated mathematical algorithms to identify i) deletions of different lengths with increasing stretches of microhomology to 20bp, ii) small (1-3bp) insertions resulting from templated synthesis in trans previously reported in drosophila, and iii) large (>/ = 18bp) insertions resulting from templated synthesis in cis (snapback synthesis) described in mouse embryonic stem (ES) cells [14, 28–30]. Both types of insertions require DNA synthesis that creates microhomologies through DNA secondary structures, followed by end-joining, DNA extension, processing, and ligation.

**Fig 4:**
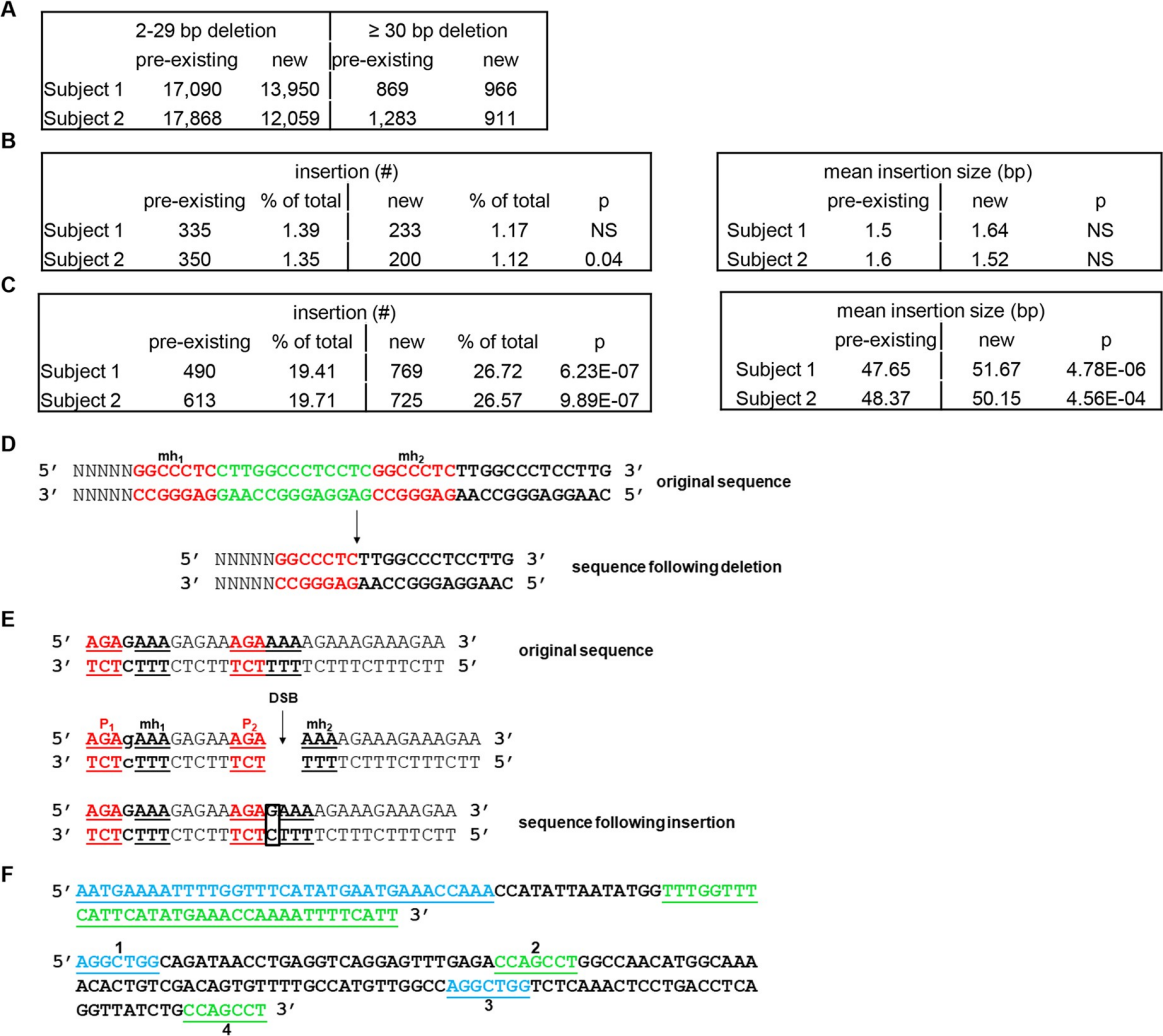
EBV-transformed cells demonstrate newly-generated deletions and insertions bearing signatures of MMEJ. Two-week old LCL derived from 2 healthy subjects and their respective primary B lymphocytes were subjected to whole genome sequencing. (A, D) Newly-generated versus pre-existing deletions of different lengths bearing MMEJ-signatures are shown in A with an example of one such deletion shown in D. (B, E) Newly-generated versus pre-existing small insertions bearing signatures of synthesis-dependent MMEJ are tabulated on the left with mean insertion sizes on the right in B. An example of such an insertion with flanking regions of microhomology (mh1, mh2, P1, and P2) is shown in E; the inserted nucleotide is boxed. In this example, looping-out of the top strand and mispriming of P2 on P1 of the bottom strand is followed by insertion of a single nucleotide (G) and templated synthesis of mh2; the strands then separate and resume DNA synthesis following realignment of P1, mh1, P2, and mh2 at the appropriate regions on the complementary strand. (C, F) Newly-generated versus pre-existing large insertions bearing signatures of snapback synthesis MMEJ are tabulated on the left with mean insertion sizes on the right in C. Two examples of such large insertions are shown in F. The top sequence is an example in which there is likely to have been templated synthesis through a snapback mechanism on the same strand generating 35 nucleotides of inverted repeats (underlined) resulting in an 84 nucleotide insertion. The lower sequence is a 130 nucleotide insertion in which there were multiple snapback events resulting in two sets of inverted repeats of 7 nucleotides each (numbered 1–4). #2 resulted from using #1 as a template or another 7-mer matching #1 in the original sequence. Similarly, #3 resulted from using #2 (or another 7-mer matching #2 in the original sequence) and #4 resulted from using #1 or #3 as a template (or another matching 7-mer in the original sequence). The intervening sequences likely arose from a combination of non-templated insertions and insertions templated from complementary regions in the original sequence.

Comparison of LCL genomes to genomes of their respective primary B lymphocytes revealed that substantial numbers of both small (2-29bp) and large (>/ = 30bp) deletions existed in B cells prior to viral transformation with new deletions of both types generated following transformation of cells from both subjects (Fig 4A and 4D). Newly-generated small deletions were more abundant than large deletions (Table 1). Still, the fraction of large deletions resulting from longer stretches of microhomology were higher in newly-transformed cells compared to similar pre-existing deletions (Table 2). While there were newly-generated small insertions (<4bp), their frequency and average size remained unchanged (Fig 4B and 4E). In contrast, there were more newly-generated large insertions (>17bp) that were also longer compared to pre-existing ones (Fig 4C and 4F). Collectively, EBV-transformed cells with constitutively active STAT3, though deficient in HR, competently repair DSBs via MMEJ, rapidly accrue genome-wide scars from MMEJ-mediated deletions and insertions, and succumb to PARP inhibition.

**Table 1:** Characteristics of short deletions (2-29bp).

LCL	minimum mh	pre-existing deletions	with mh	fraction with mh	new deletions	with mh	fraction with mh	p
	(bp)			(%)			(%)	
1	2	17090	13190	0.77	13950	11710	0.84	5.58E-50
	3	12415	9199	0.74	9294	7295	0.78	7.15E-14
	4	10613	7485	0.7	7342	5381	0.73	5.74E-05
	5	7842	5342	0.68	5673	3959	0.7	0.04
	6	6965	4697	0.67	5011	3385	0.68	NS
	7	5698	3776	0.66	4211	2773	0.66	NS
	8	5189	3366	0.65	3813	2438	0.64	NS
	9	4043	2513	0.62	3085	1880	0.61	NS
	10	3680	2221	0.6	2770	1654	0.6	NS
	11	3071	1790	0.58	2375	1376	0.58	NS
	12	2857	1596	0.56	2167	1230	0.57	NS
	13	2188	1149	0.53	1776	949	0.53	NS
	14	2024	1020	0.5	1639	843	0.51	NS
	15	1682	837	0.5	1429	716	0.5	NS
	16	1515	708	0.47	1283	624	0.49	NS
	17	1176	497	0.42	1039	474	0.46	NS
	18	1096	443	0.4	971	417	0.43	NS
	19	932	346	0.37	793	311	0.39	NS
	20	847	299	0.3	706	258	0.37	NS
2	2	17868	13570	0.76	12059	10146	0.84	1.01E-65
	3	13293	9666	0.73	7760	6104	0.79	9.56E-22
	4	11493	7889	0.7	6071	4505	0.74	1.63E-14
	5	8680	5723	0.66	4686	3311	0.71	2.88E-08
	6	7847	5070	0.65	4137	2838	0.69	1.28E-05
	7	6529	4172	0.64	3497	2320	0.66	0.015
	8	5962	3713	0.62	3169	2046	0.65	0.033
	9	4718	2781	0.6	2608	1623	0.62	0.006
	10	4342	2500	0.6	2380	1441	0.61	0.019
	11	3639	2017	0.55	2056	1206	0.59	0.019
	12	3372	1803	0.53	1908	1104	0.58	0.002
	13	2606	1291	0.5	1538	852	0.55	0.0003
	14	2408	1136	0.47	1419	773	0.54	1.50E-05
	15	2044	928	0.45	1240	656	0.53	3.55E-05
	16	1824	779	0.43	1123	572	0.51	1.60E-05
	17	1461	576	0.39	938	448	0.48	6.72E-05
	18	1333	481	0.36	862	396	0.46	5.14E-06
	19	1099	369	0.34	732	318	0.43	2.42E-05
	20	1005	309	0.31	671	263	0.39	0.0004

**Table 2:** Characteristics of long deletions (>/ = 30bp).

LCL	minimum mh	pre-existing deletions	with mh	fraction with mh	new deletions	with mh	fraction with mh	p
1	(bp)	869		(%)	966		(%)	
	2		664	0.76		761	0.79	NS
	3		622	0.72		714	0.74	NS
	4		559	0.64		662	0.69	NS
	5		542	0.62		640	0.66	NS
	6		531	0.61		620	0.64	NS
	7		516	0.59		601	0.62	NS
	8		500	0.58		581	0.6	NS
	9		483	0.56		558	0.58	NS
	10		465	0.54		533	0.55	NS
	11		452	0.52		515	0.53	NS
	12		437	0.5		495	0.51	NS
	13		418	0.48		476	0.49	NS
	14		403	0.46		462	0.48	NS
	15		385	0.44		443	0.46	NS
	16		361	0.42		425	0.44	NS
	17		332	0.38		411	0.43	NS
	18		311	0.36		392	0.41	0.039
	19		295	0.34		379	0.39	0.02
	20		276	0.32		364	0.38	0.009
2		1283			911			
	2		958	0.75		702	0.77	NS
	3		895	0.7		668	0.73	NS
	4		822	0.64		611	0.67	NS
	5		796	0.62		591	0.65	NS
	6		759	0.59		576	0.63	NS
	7		721	0.56		556	0.61	0.026
	8		702	0.55		537	0.59	NS
	9		677	0.53		518	0.57	NS
	10		651	0.51		503	0.55	0.042
	11		632	0.49		484	0.53	NS
	12		600	0.47		463	0.51	NS
	13		576	0.45		454	0.5	0.024
	14		553	0.43		444	0.49	0.01
	15		526	0.41		432	0.47	0.003
	16		502	0.39		410	0.45	0.006
	17		474	0.37		391	0.43	0.005
	18		443	0.35		376	0.41	0.001
	19		417	0.33		361	0.4	0.0006
	20		395	0.31		341	0.37	0.001

### A STAT3-gene signature to predict susceptibility of cancers to PARP inhibition

**Fig 5:**
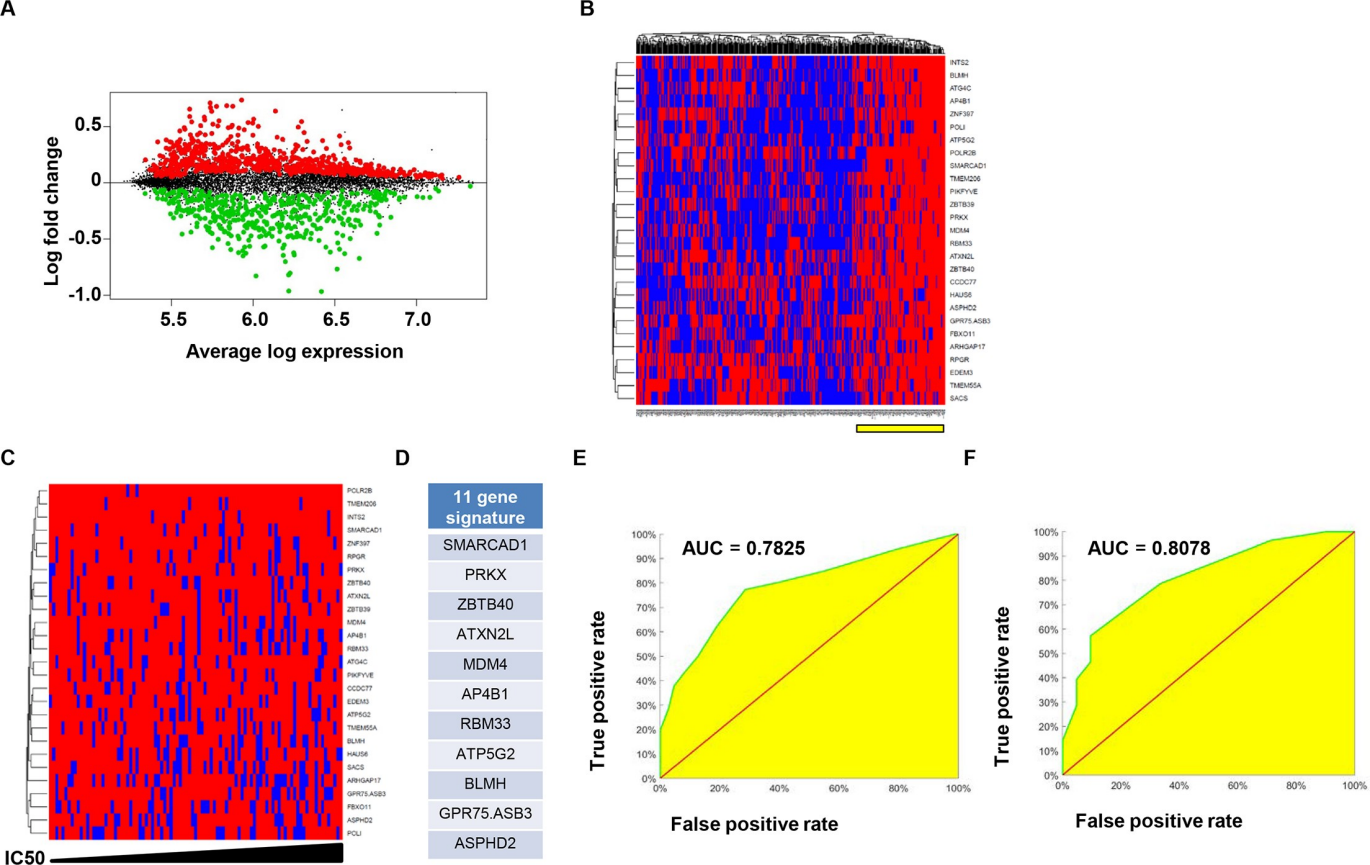
Cross-analysis between STAT3-targetome, gene expression, and PARP inhibitor susceptibility data in cancer lines from a range of tissues identifies a gene signature that predicts susceptibility to PARP inhibition. (A) Mean-difference plot showing differential expression of STAT3 transcriptional targets between cancer lines with highest sensitivity (corresponding to ~30% of sensitive lines) and those with highest resistance (corresponding to ~10% of resistant lines) to a PARP inhibitor. Red spots represent 699 genes with significantly higher expression in highly sensitive lines, green spots correspond to 472 genes demonstrating higher expression in highly resistant lines, and black spots represent 5899 genes that were not differentially expressed. (B and C) Shown in B is a hierarchically clustered binary plot of expression of 27 (of 699) genes with higher expression in all lines with high sensitivity to PARP inhibitor; high or low calls were based on whether expression exceeded the sensitive mean minus one standard deviation. A second binary plot, derived from the plot in B, displayed on an IC50 scale using the subpopulation of lines (indicated by a yellow bar in B) that expressed overall high levels of the 27 genes is shown in C. Examination of this binary plot led to the selection of nine genes with high expression in lines with low IC50s (i.e. in sensitive lines) but low expression in lines with high IC50s (i.e. in resistant lines). Two additional genes found to be good predictors of IC50 based on independent Lasso and Elastic net analyses of STAT3-transcriptional targets were also among the 27 genes from above. These were added to the nine genes to yield an 11-gene signature, shown in D. (E and F) ROC curves derived from applying the 11-gene signature to experimental data on gene expression and susceptibility to PARP inhibitors in all cancer lines (>450 from a variety of tissue types; E) versus blood cancer lines (F) within the Cancer Genome Project dataset; AUC, Area under the ROC Curve.

With STAT3 now linked to HR impairment and *BRCA*ness in EBV-transformed cells and also known to be constitutively active in a variety of cancers [31], we asked if a STAT3-gene signature could predict susceptibility of cancers to synthetic lethal approaches. We therefore performed a cross-analysis between transcriptomic and PARP inhibitor susceptibility data from 452 cancer lines derived from a wide variety of tissues archived by the Cancer Genome Project (Wellcome Trust Sanger Institute, UK), and a publically-available STAT3 ChIP-seq dataset from human cells by the ENCODE Project (NHGRI, USA) [32–34]. By comparing lines that were highly-sensitive to PARP inhibition to those that were highly-resistant, we identified 27 STAT3-target genes that were upregulated in all highly-sensitive lines (Fig 5A).

Examination of expression of the 27 genes on hierarchically clustered binary plots (Fig 5B and 5C) resulted in identification of 9 genes with high expression in lines with low IC50s (i.e. in sensitive lines) but low expression in lines with high IC50s (i.e. in resistant lines). In parallel, Lasso and Elastic net regression analyses were performed to identify four STAT3-target genes that were common between 3 models and the original 27 genes from above. Two of the four genes were distinct from the 9 gene subset. Together, they yielded a set of 11 genes (Fig 5D). We then tested the performance of the STAT3 11-gene signature using all cell lines in the database with experimental data on gene expression and susceptibility to PARP inhibitors. Our analysis revealed a ROC curve with AUC of 0.7825 (Fig 5E). Since our experimental findings originated in lymphocytes, we also tested the signature on all blood cancer lines in the database and found that the AUC was 0.8078 (Fig 5F). Thus, a small set of STAT3-target genes predicts susceptibility of EBV-unrelated cancer cell lines including blood cancer lines to PARP inhibition.

## Discussion

Our findings mechanistically link constitutively active STAT3 to HR impairment in EBV-transformed cells and support the idea that EBV-lymphomas including LPD, BL, and certain DLBCL (Diffuse Large B-Cell Lymphomas) may be susceptible to synthetic lethal approaches including PARP inhibition. With a large number of human cancers, both EBV-related [35, 36] and unrelated [31], demonstrating constitutively active STAT3, the predictive STAT3-gene signature also opens the possibility of personalizing synthetic lethal therapy for patients with such STAT3^hi^ cancers. Because STAT3 is constitutively active in other EBV-mediated diseases such as chronic active EBV infection (CAEBV) [37], by extension, synthetic lethal approaches may also be an option for CAEBV. While *BRCA*ness is known to arise from inactivating mutations or epigenetic silencing of HR-related genes [21, 22], our findings are particularly germane in view of clinical observations that many cancer patients without detectable mutations in genes encoding HR components such as *BRCA* genes also derive significant clinical benefit from PARP inhibitors [38, 39].

Viruses have been instructional in shaping our understanding of basic biological processes. This is another example that now demonstrates that through EBV-mediated activities, HR may be impaired–not by influencing HR proteins themselves but by altering post-translational modifications of upstream mediators such as STAT3 and Chk1. In doing so, these findings also tie STAT3 to HR. Recently, β-HPV E6 was also found to impair phosphorylation of Chk1. While this resulted in HR impairment, the mechanism that impaired ATR’s ability to phosphorylate Chk1 remains unclear [40]. In the case of EBV, we have shown that active STAT3 transcriptionally upregulates the anti-apoptotic form of caspase 9 which then activates caspase 7 resulting in degradation of claspin, a key adaptor protein [9–11]. In the absence of claspin, ATR, though activated by viral oncoprotein-induced replication stress, is unable to phosphorylate Chk1. Consequently, EBV’s disruption of Chk1 phosphorylation ensures unhindered passage of infected/transformed cells through the cell cycle. That said, whether EBV’s impact on HR is an intended or unintended consequence remains unclear. Certainly, shifting the burden of DNA repair towards more error-prone mechanisms like MMEJ adds to the mutation load and genomic instability in EBV-transformed cells.

While available tests that screen for HR function and known HR mutations or silencing mechanisms already do not adequately predict susceptibility to synthetic lethal therapies, our findings make it even harder for existing tests to predict which individual cancers may respond to synthetic lethal therapies. There is a recognized need for biomarkers that predict responses to synthetic lethal strategies such as PARP, ATM, and ATR inhibitors. Currently, HR-related mutation signatures including the recently published Signature 3, a few gene expression profiles applicable to breast and ovarian cancers, and a small number of HR assays are available for prediction of susceptibility to PARP inhibitors [41–45]. However, these are not yet completely inclusive of responders [38]. Of the 11 genes indicative of susceptibility to PARP inhibition in our study, five are directly or indirectly linked to DNA repair or DNA damage signaling. SMARCAD1 was recently shown to mediate DNA end resection at DSBs for HR-mediated repair [46]. PRKX encodes a serine threonine protein kinase that phosphorylates MBD4/MED1, a DNA N-glycosylase involved in mismatch repair [47]. MDM4/MDMX is known to regulate p53 and p73 and is itself regulated via phosphorylation by ATM, Chk1, and Chk2 [48]. BLMH is a DNA-binding cysteine peptidase that mediates Bleomycin resistance [49]. ZBTB40 is a zinc finger protein whose function is presently unknown; however, on a proteomic analysis, it was a target of phosphorylation by ATM/ATR in response to DNA damage [50]. Little is known about the function of five other genes (ATXN2L, RBM33, ATP5G2, GPR75.ASB3, and ASPHD2). The last, AP4B1, is a protein that regulates vesicular transport of proteins [51]. While the 11 genes predict susceptibility to synthetic lethal therapies, at this time there is no evidence that they contribute to susceptibility of cancer cells to such therapies.

Our findings also provide evidence for robust contribution by MMEJ to DNA repair in EBV-transformed human B cells with active STAT3 - a contribution that rapidly results in the accumulation of deletions as well as insertions, both small and large. Based on the cell types that we used, we believe that these findings should apply to EBV-LPD, BL, and DLBCL harboring EBV in type III latency. Whether MMEJ-derived deletions and insertions exist genome-wide in other EBV- and non-EBV related cancers remains to be seen. MMEJ-mediated repair is mostly believed to function as a back-up mechanism when conventional forms of DSB repair i.e. HR and NHEJ are not available [26]. Indeed, generation of large deletions with signatures of microhomology in HR-deficient LCL is in line with the observation of large deletions (>50bp) with microhomologies in breast, ovarian, and pancreatic cancers with *BRCA* mutations [52]. This concordance would support the idea that cancers bearing large deletions with signatures of MMEJ-mediated repair are more likely to be susceptible to PARP inhibition. In contrast to deletions, evidence for insertions that typically require DNA synthesis is presently based on experimental systems using drosophila and mouse embryonic stem cells [28–30]. Our findings demonstrate that not only do such insertions exist genome-wide in transformed human cells but that they can accumulate rapidly. Moreover, like large deletions, large insertions (with longer regions of microhomology) were more prevalent in newly-transformed cells than in primary B cells.

While both deletions and insertions with microhomology were also identified in primary B cells, in contrast to transformed cells, those in primary B cells arose over the lifetime of each individual. Notably, very few insertions and deletions in transformed or primary B cells were within Ig genes; this was not surprising since Ig gene recombination primarily uses classical NHEJ. Importantly, however, the existence of MMEJ-signatures in pre-existing deletions and insertions in primary B cells hints at roles for MMEJ-mediated repair in physiologic contexts. This last is supported by a report of structural variants in human genomes that likely arose from MMEJ [14, 26].

In terms of susceptibility of EBV-transformed cells to Olaparib, this drug is an inhibitor of PARP1 and 2, and MMEJ requires PARP1 to facilitate the recruitment of DNA polymerase theta to DNA lesions [26]. Although this would suggest that susceptibility of EBV-transformed cells and lymphoma cells to Olaparib was a result of blocking MMEJ, additional contribution via impairment of other mechanisms such as base excision repair which uses PARP1-3 cannot be excluded. Regardless of other potentially targetable mechanisms, our findings indicate that EBV-lymphomas may be effectively treated with Olaparib, a strategy that has not been previously considered.

## Materials and methods

### Study subjects and ethics statement

Blood was obtained from study subjects following informed consent. The study of human subjects was approved by the Institutional Review Boards at the University of Florida, Stony Brook University, and the NIAID. Written informed consent was obtained from study subjects. Healthy EBV-seronegative volunteers ranged from 18 to 28 years of age. Peripheral blood B cells were isolated and EBV-LCL were derived from three healthy subjects and three Job’s syndrome patients. All except EBV-LCL derived from two healthy subjects were previously described [10].

### Isolation of primary B lymphocytes and infection with EBV

Peripheral blood B cells were isolated by negative selection and infections with EBV were performed as described [9].

### Culture conditions

Newly-infected B cells and previously established EBV-LCL were grown in culture using conditions described [9]. For experiments using AG490 and Olaparib, drug was supplemented at the initial concentration every fourth day. For experiments using Bleomycin, the drug was added for an hour, following which cells were washed and placed back in culture. We had previously demonstrated 50μM AG490 to be minimally toxic to EBV-infected B-cell lines [9, 10].

### Antibodies

The following primary antibodies were used for immunologic applications: rabbit anti-human STAT3, rabbit anti-human pSTAT3 (Y705), mouse anti-human RAD51, rabbit anti-human pATR (S428), mouse anti-human γH2AX, mouse anti-human β-actin, rat anti-(EBV)EBNA2 (clone R3) [53]. Secondary antibodies included HRP-anti-mouse Ab, HRP-anti-rabbit Ab, FITC-anti-mouse IgG, PE-anti-rabbit IgG, and PE-anti-rat IgG.

### Flow cytometry

For assessment of cell-cycle distribution, cells were fixed, permeabilized, and stained with anti-EBNA2 antibody and 50μg/ml propidium iodide supplemented with 1μg/ml RNase A, as previously described [34]. For DR-GFP and EJ2-GFP assays, cells were transfected with the appropriate combinations of plasmids and harvested 48 hours later. Data were acquired using a FACS Calibur and analyzed using FlowJo software.

### Immunofluorescence microscopy

Cells were stained as for flow cytometry, washed, cytospun onto glass slides, air dried, and mounted with DAPI Prolong Gold Anti-fade (Life Technologies). Images were acquired at 40× magnification on an AxioScope A1 microscope (Zeiss) with SPOT v4.0 software. When counting cells with nuclear foci, images were blinded and counted by two individuals; only nuclei with ≥5 foci were considered positive.

### Immunoblotting

Total extracts from 1x10^6^ per mL cells were analyzed by immunoblotting as described [34].

### Plasmids, siRNAs, and transfections

Plasmids DR-GFP, pCBASce (encoding I-Sce1 enzyme), and pCAGGS were gifts from Dr. Maria Jasin [17]. EJ2-GFP-puro was a gift from Dr. Jeremy Stark (Addgene plasmid # 44025) [27]. Plasmids bearing wild-type and phosphorylation site Chk1 mutant S345A were gifts from Dr. Kum Kum Khanna [54]. BL cells and EBV-LCL were transfected using an Amaxa II nucleofector with plasmids or siRNA [targeting STAT3 (sc-29493) or scrambled (sc-37007), Santa Cruz Biotechnology] as previously described [10].

### MMEJ analysis of whole genomes

Sample sequencing was outsourced to BGI (www.bgi.com) who used a standard bioinformatics pipeline for indel detection, as follows. FASTQ files were first preprocessed for quality control: 1) removing reads containing sequencing adapters, 2) removing reads where more than 50% of base calls were low-quality, defined as Q< / = 5, and 3) removing reads with more than 10% base calls having N, which together resulted in >97% Q20 reads for Read 1 and >93% Q20 reads for Read 2. A further quality control step was implemented at the variant call level (see below). Burrows-Wheeler Aligner (BWAV0.7.12) was used for mapping to the human reference genome (GRCh37/hg19). Mapping was performed for each lane separately using the BWA-MEM method. Duplicate reads were removed using Picard-tools (v1.118). Local realignment, which realigns reads to minimize mismatches across all the reads (indels often lead to many bases mismatching the reference genome near the misalignment), was performed using the GATK (v3.3.0) commands RealignerTargetCreator and IndelRealigner. Base quality score recalibration (BQSR), a standard step which adjusts read quality scores to deal with systematic technical error, was performed using GATK BaseRecalibrator and indel calls were made using GATK HaplotypeCaller. Variant Quality Score Recalibration (VQSR), a standard method for assigning a well-calibrated probability to each variant call, was used to score and filter the raw variant callset (GATK commands: SelectVariants, VariantRecalibrator and ApplyRecalibration). Variant calls were further filtered for quality such that any calls having the VCF field QUAL<40, were removed.

Raw sequence data are available in the SRA database with the accession numbers SRR12374618, SRR12374619, SRR12374620, and SRR12374621.

### MMEJ–Deletion analysis

Custom R scripts were used to implement the analysis, as follows. Any indels previously identified by the 1000 genomes, ESP6500 or dbSNP141 were removed, as were any that were shared between the two human samples, which we assumed to be spurious. For analysis of deletions with microhomology, the sequence adjacent to each deletion was extracted based on the human genome (R package BSgenome.Hsapiens.UCSC.hg19). For analysis of “small” deletions, deletions of at least 2bp and less than 30bp were evaluated (Table 1), and for “long” deletions, those between 30bp and 500bp were evaluated (Table 2). In each case, the number of contiguous matching nucleotides between the deletion and the adjacent sequence (counted from the beginning of each sequence) was recorded for each subject and sample, B-cell or LCL. Different microhomology length thresholds (ranging from 2 to 20bp) were then used to filter these results and distinct sets of deletions were constructed representing (A) the intersection of B-cell and LCL samples, which were assumed to have occurred at the B-cell stage and (B) those deletions unique to LCL samples, assumed to have arisen in LCLs. For a given subject and size threshold *T*, the deletions in each of these datasets were counted and separated into those having a (deletion vs adjacent sequence) match of at least length *T*, and those not having the match. For each threshold considered, any deletions having length less than T were not considered since these could not possibly match. Contingency tables were constructed comparing the proportion of matches in the intersection set to those in the LCL-unique set. A chi-squared test (as implemented in the R function prop.test) then used the contingency table to assess whether the proportion of matches, representing MMEJ events, was significantly changed in the LCL-unique set compared to the intersection. This analysis was performed separately for each subject.

### MMEJ–Small insertion analysis

To detect synthesis-dependent small insertions, we extracted the surrounding sequence context (+/-15nt) surrounding the insertion (from the hg19 human genome) and identified whether there was a repeated sequence either 5’ or 3’ to the insertion that matched the full insertion and also at least 2nt on either side. Matches also needed to have a gap of at least 1nt between the two sequences. As shown in the example in Fig 4E, the repeated sequence 5’-AGAgAAA-3’ is found upstream of the insertion-containing counterpart which contains P2 (AGA), the insertion (G) and mh2 (AAA). Also both P2 and mh2 are of length>/ = 2 and the two sequences are >/ = 1nt apart. Using this method, all insertions in each sample were thus labeled as small insertions, or not.

### MMEJ–Large insertion analysis

For synthesis-dependent snapback (large) insertions, only the insertion sequence was considered. Each sequence was compared to its own reverse-complement sequence and any repeats of >/ = 7nt also separated by at least 4nt were labeled as snapback-like (see examples in Fig 4F). To avoid spurious false negatives, only insertions of at least (2×7) + 4 = 18nt were considered for this analysis. All such insertions in each sample were labeled as snapback-like, or not.

As with the analysis of the deletions described above, for both small and large insertions, contingency tables were constructed comparing the proportion of matches in the intersection set to those in the LCL-unique set. Chi-squared tests were again used to compare the LCL-unique set to the intersection for each of the two samples.

### Analysis of cancer lines

Gene expression data from 452 cancer lines from a variety of tissue types from the Cancer Genome Project were examined; data were previously normalized using robust multi-array averaging [33]. Differential gene expression was examined between cancer lines that were highly-sensitive (18 lines; corresponding to ~30% of sensitive lines) and highly-resistant (23 lines; corresponding to ~10% of resistant lines) to PARP inhibition. We then determined which genes, predicted to be transcriptional targets of STAT3 (~8,000 genes from a publically-available STAT3 ChIP-seq) [32], were upregulated in the highly-sensitive lines compared to the highly-resistant lines using limma-voom [55] which estimates precision weights for linear modelling in the empirical Bayesian analysis pipeline and results in moderated t-statistics. Adjusted p-values were calculated and filtered using a false-discovery rate of 0.05. There were 699 differentially expressed genes upregulated in the highly-sensitive lines. Of the 699 genes, 27 were upregulated in all highly-sensitive lines relative to the mean resistant expression.

A hierarchically clustered binary plot of expression data of the 27 genes in all cell lines was generated using high or low calls that were determined based on whether expression exceeded the sensitive mean minus one standard deviation or not. A second binary plot was generated on an IC50 scale using the subpopulation of lines (indicated by a yellow bar; Fig 5B) that expressed overall high levels of the 27 genes. Of these, nine genes with high expression in lines with low IC50s (i.e. high expression in sensitive lines) but low expression in lines with high IC50s (i.e. low expression in resistant lines) were selected.

In parallel, Lasso (120 steps with 5-fold cross validation) [56] and Elastic net [57] analyses were run in SAS on the 8,000 STAT3-transcriptional targets using five distinct modeling parameter sets (5-fold 120-steps, 5-fold 500-steps, 10-fold 120-steps, 10-fold 500-steps) where the gene expression for the STAT3-transcriptional targets was used to predict IC50. All models performed similarly based on gene sets selected and root mean-squared error. From these analyses, four predictive genes were identified in common between the three models run for 120 steps and the 27 gene set from above. Two of these genes, which were good predictors of IC50, were distinct from the nine gene subset from above. These were added to the nine to make a total of 11 genes.

For ROC curves, samples were binned by IC50 from zero to seven by 0.5 intervals individually for primarily red (i.e. lines expressed at overall high levels) and mixed zones as determined from the binary heatmap (Fig 5B) where zones were delineated such that at least 60% in Fig 5E or 82% in Fig 5F of the genes were expressed at high level (red) or not (mixed). The percentage of samples falling into each bin were plotted in scatter plots with mixed zone percentages on the x-axis and red zone percentages on the y-axis. The trapezoidal rule was used to estimate the area under the curve (AUC).

### Statistical analysis

Unless described otherwise (for MMEJ analysis and analysis of transcriptomic datasets in cancer lines), statistical significance was determined using p values that were calculated by comparing the means of two groups of interest using unpaired Student t test.
